# Move for Life an intervention for inactive adults aged 50 years and older: a cluster randomised feasibility trial

**DOI:** 10.3389/fpubh.2024.1348110

**Published:** 2024-05-15

**Authors:** Catherine B. Woods, Andrew O’Regan, Ciaran Doyle, Grainne Hayes, Amanda Clifford, Alan E. Donnelly, Paddy Gillespie, Liam Glynn, Andrew W. Murphy, Ali Sheikhi, Enrique García Bengoechea

**Affiliations:** ^1^Physical Activity for Health Research Centre, Department of Physical Education and Sport Sciences, University of Limerick, Limerick, Ireland; ^2^Health Research Institute, University of Limerick, Limerick, Ireland; ^3^School of Medicine, University of Limerick, Limerick, Ireland; ^4^Ageing Research Center, School of Allied Health, University of Limerick, Limerick, Ireland; ^5^Centre for Research in Medical Devices (Cúram) and Health Economics and Policy Analysis Centre, NUI Galway, Galway, Ireland; ^6^HRB Primary Care Clinical Trials Network, Discipline of General Practice, NUI Galway, Galway, Ireland

**Keywords:** community-based, older adults, physical activity, energy expenditure, body composition, physical function, well-being

## Abstract

**Background:**

Move for Life (MFL) is a theory-informed intervention that was developed to augment established physical activity (PA) programmes and enable inactive adults aged 50 years and older to be more active. This study examined the feasibility of MFL and sought to provide evidence of its potential for improving PA and associated health outcomes.

**Methods:**

A 3-arm cluster randomised feasibility trial compared MFL intervention, usual provision (UP) and control (CON) groups at baseline (T0), post-intervention (T1, at 8, 10 or 12- weeks) and 6-month follow up (T2). We used purposive sampling strategies to recruit participants according to characteristics of interest. Feasibility outcomes assessed recruitment, fidelity, adherence, retention and data completion rates based on pre-set criteria. Primary outcomes were accelerometer-based moderate-to-vigorous intensity PA (MVPA) and self-reported compliance with physical activity guidelines (PAGL). Secondary outcomes included light intensity PA (LiPA), standing time, sedentary time, body composition (adiposity), physical function and psychological well-being. We used linear mixed models (continuous outcomes) or generalized estimated equations (categorical outcomes) to estimate group differences over time in the study outcomes.

**Results:**

Progression criteria for feasibility outcomes were met, and 733 individuals were recruited. Considering a 6-month period (T0-T2), while self-reported compliance with PAGL increased in MFL relative to UP and CON and in UP relative to CON, standing time decreased in MFL relative to CON and sedentary time increased in the latter compared to UP. Waist circumference decreased in MFL relative to UP and CON. MFL outperformed UP in the Timed Up and Go Test while MFL and UP increased the distance covered in the Six-Minute Walk Test compared to CON. Psychological well-being increased in MFL relative to CON (all *p* < 0.05).

**Conclusion:**

Findings show that MFL is feasible, while data are promising with regards to the potential of improving community PA programmes for adults aged 50 or more years.

**Clinical trial registration:**

https://www.isrctn.com/Registration#ISRCTN11235176.

## Introduction

The pace of population ageing is increasing much faster than in the past. According to recent estimations, by 2030, 1 in 6 people in the world will be aged 60 years or over^1^. An ageing society coupled with physical inactivity has led to an increase in the incidence of non-communicable diseases (NCD), much of which occur prematurely in adults, while the associated mortality rate rises (1). The Health and Positive Aging Initiative (HaPAI) was established by the Department of Health in Ireland to research how best to support active ageing, health, and well-being of Irish adults aged 50 years and older (2). Its objective is to inform the Department’s policy response to meeting the challenges associated with healthy population ageing. HaPAI emphasises the role of modifiable lifestyle behaviours, in particular, the importance of regular health enhancing physical activity (PA) especially for inactive adults as they age. However, evidence from a global review identified that 45% of adults aged over 60 years were inactive (3). Likewise, several studies conclude that adults 50 years and older would benefit from more PA (4). In Ireland, trends are considerably worse with 65% of 55–64 year-olds and 82% of those aged over 75 years not meeting the physical activity guidelines (PAGL) (5) of at least 30 min of moderate intensity activity on 5 days a week or 150 min weekly (6).

PA, defined as any bodily movement produced by the skeletal muscles that requires energy expenditure greater than that at rest (7), is associated with a range of positive physical and psychosocial outcomes. Systematic reviews in older adults have found that PA can improve performance of activities of daily living (8), gait speed (9), balance (8), physical function (9), and risk of falls leading to medical care (10, 11). In addition, PA has been found to prevent social isolation and loneliness (12) and reduce depressive symptoms (13, 14). Evidence also suggests that low-dose moderate to vigorous PA (MVPA), below current guidelines, reduces mortality in adults aged 60 years and over (15). MVPA is defined based on the intensity of the activity and typically requires individuals to use at least three (moderate) or six (vigorous) times as much energy per minute as they would do when sitting quietly (16). Other studies show that additional forms of activity related energy expenditure (i.e., LiPA, standing) or reducing sedentary time, or both, are associated with health benefits (17–19).

Community programmes have the potential to expand population reach and have been shown to promote PA across the lifespan (20), while there is evidence that complex interventions including behavior change strategies can both increase (21) and maintain (22) PA levels of adults aged 55 years and over. Community-based interventions are appealing as they are accessible to people in their social or geographical area (23), usually target all groups in a particular community (24), and can be effective in overcoming commonly cited PA barriers (25, 26). In addition, multi-component interventions with peer-led elements are also proven to be effective in promoting health behavior change, including PA in adults, though such approaches can be expensive (27–29). Intervention development and implementation are necessary to address the inactivity challenge, particularly prevalent in older adults in Ireland.

Despite the fact that many countries, including Ireland, adopted national policies or action plans to increase PA, a series published in 2016 in Lancet describe their implementation as weak (30). The authors concluded that the greatest progress is likely to occur through interventions that are effective in promoting PA, implemented at scale, regularly assessed, and fully embedded within an enabling system (30). In order to contribute to this effort, in collaboration with relevant stakeholders, we developed a pragmatic intervention (Move for Life, MFL) informed by several sources of evidence to reach and help inactive adults aged 50 years+ increase their PA. In line with the HaPAI objectives, the intervention was designed to fit within existing group-based structured PA programmes delivered by state- funded Local Sports Partnerships in Ireland, thus maximising the likelihood of translation of findings into policy recommendations and ultimately practice. When properly designed, feasibility studies can provide valuable insights into intervention development, study methods, preliminary intervention effects, implementation strategies, and opportunities for refinement and optimization (31, 32). This study aimed to examine the feasibility of the MFL intervention and its potential on activity related energy expenditure and associated health outcomes for inactive adults aged 50 or more years over a 6-month period.

## Methods

The protocol for the MFL study is available elsewhere (33).

### Setting

The trial took place in the Health Service Executive Mid-West region of Ireland in pre-existing Local Sports Partnership (LSP) community sport and PA hubs that were developed as part of Ireland’s National Physical Activity Plan and whose purpose is to increase engagement in physical activity generally and particularly amongst disadvantaged, marginalised and hard to reach groups. LSPs are state-funded community-based organisations whose purpose is to provide structured sport and physical activity programmes and opportunities for the communities they serve, often using trained instructors (tutors). Eight hubs across counties Clare (*n* = 4) and Limerick (*n* = 4) were recruited. Hub inclusion criteria required professional expertise to run four nationally approved PA programmes suitable for inactive middle-aged and older adults. These were *Men on the Move* [an evidence-based mixed sport programme for men; 12 weeks, 2 sessions/week (34)]*, Women on Wheels/Bike for Life* (a ‘Get Ireland Cycling’ cycling programme; 10 weeks, 1 session/week)*, Go for Life* (an indoor mixed games programme developed by ‘Age and Opportunity’, the national organization working to enable the best quality of life for Irish adults as they age; 8 weeks, 1 session/week) *and Get Ireland Walking* (an outdoor community walking programme; 10 weeks, 1 session/week). In total, 32 freely accessible PA programmes were implemented over the trial period.

### Study design

A cluster design was used to overcome contamination problems and LSP hubs were defined as the units of randomisation (the clusters). Participants within these hubs (units of analysis) were randomised to one of the three arms, (i) the MFL intervention group (MFL; the existing PA programmes plus the MFL augmentation, 3 hubs); (ii) the usual provision (UP; the existing PA programmes consisting of PA classes delivered as normal, 3 hubs); and (iii) the control group (CON; information on PA only, 2 hubs). CON individuals were invited to participate in the PA programmes once the trial was completed. Each hub was geographically separated to reduce the possibility of contamination and clusters were stratified as rural or urban. Randomisation of hubs occurred following baseline assessment and was conducted by an external researcher (JN), using a process of minimisation (35).

### Intervention

The MFL intervention is described in detail elsewhere (36). In brief, MFL aimed to enhance the impact of established national PA programmes by augmenting the professional model (PA tutors), through a multimodal intervention. LSP tutors, professionally trained in the delivery of the PA programmes mentioned above, received twice 3-h workshops designed to provide them with training materials in behavioral theory: social cognitive theory (37), self-determination theory (38), and principles of group dynamics applied to PA and exercise settings (39) and how to embed behavior change techniques into their programmes. Consistent with these conceptual frameworks, behavior change techniques were grouped into strategies to help participants develop cognitive and behavioral skills to manage their PA behavior, give and receive social support from other participants and programme tutors, and develop group attitudes and norms conducive to group integration/cohesion. Additionally, the tutors identified suitable class members who volunteered to become peer-mentors and attend a 3-h workshop to enhance PA programme peer support, sustain both group and individual long-term PA and scope out useful services provided by their LSP. MFL handbooks supported the training with a tutor protocol for the delivery type, frequency and intervention content, and a paired participant handbook with session specific information, individual and group tasks. Training was tailored to meet group and individual needs and supported by a MFL researcher who assisted tutors and peer mentors throughout the study period. The PA programmes and intervention took place from 2018–2019.

### Procedures

A diverse range of purposive recruitment strategies informed by our published qualitative research were used (40). Individuals who expressed an interest attended a ‘health check appointment’ where they were informed about the study in person, and in writing, and provided informed consent as per ethic’s committee approval (University of Limerick, Faculty of Education and Health Sciences Research Ethics Committee, 2018_02_15_EHS). Consenting individuals that met inclusion criteria as per the study protocol completed baseline measures and their hubs were subsequently assigned to the CON, UP or MFL arm. To be included in the trial, participants had to be inactive based on a self-report screening measure described in the study outcomes section below, community dwelling, aged 50 years and over, and able to exercise independently. Participants were excluded if they were aged under 50 years, active (according to the self-report screening measure), and unable to exercise independently. Outcome measures were collected at baseline (T0), post-intervention (T1, at 8, 10 or 12- weeks), and 6-month follow up (T2).

### Feasibility outcomes

The feasibility outcomes assessed against pre-set progression criteria were recruitment, allocation, adherence, fidelity and retention (41, 42). Recruitment data were gathered via a process evaluation questionnaire. Adherence criteria was assessed by tutor logs and validated by participant self-report at T1. Fidelity to prescribed MFL intervention content was assessed weekly by tutor fidelity checklists, monitored by a MFL researcher with phone calls, while average compliance was calculated. Tutor recruitment of peer mentors was evidenced by numbers completing MFL training. Study retention rates, data quality and adverse events, such as training related issues, e.g., muscle, tendon or joint problems, that precluded exercise participation, or other diseases that required exercise interruption, were monitored and recorded.

### Progression criteria

As per protocol, this feasibility study will progress to a full study unless there is:

- Failure by more than 40% of participants to provide reliable data for daily determination of MVPA (study design)
- Failure by more than 40% of participants to maintain engagement with the intervention (adherence)
- Failure to identify less than 80% of the required number of peer mentors by the LSP tutors in a timely fashion (fidelity).

### Primary outcomes

The primary outcomes were MVPA measured using the activPAL 3 micro accelerometer (AP3M) and compliance with PAGL measured via self-reported questionnaire. Common to all accelerometer-based outcomes in this study, participants were required to wear the AP3M device on the anterior aspect of the right mid-thigh for 24-h/day, on 8 consecutive days and were instructed to only remove the device if they were going to be submerged in water (i.e., swimming or bathing). All device removals were documented as non-wear time in a non-wear diary. MVPA was calculated using a previously developed and validated count-to-activity threshold (8,873 counts. 15 s^−1^) (43). To be included in all analyses for accelerometer-based data, participants were required to provide at least 4 days (3 weekdays, 1 weekend) of valid accelerometer data (≥ 10 h waking data/day) (44). Based on previous research in adult populations, monitor non-wear time was defined as a period of ≥60 min of consecutive zero-counts (43, 44). The total non-wear time was summed for each day and the 24-h day adjusted accordingly. For self-report, a clear definition of frequency, intensity, time and types of PA to meet PAGL was provided. In accordance with WHO guidelines (45), self-reported compliance with PAGL was assessed by the number of days over a typical or usual week that participants accumulated at least 30 min of MVPA, and if 4 days or less if they had accumulated at least 2.5 h MVPA in the last 7 days. The measure used has shown acceptable properties for classifying adults as meeting PAGL (46).

### Secondary outcomes

Secondary outcomes included accelerometer-based LiPA, standing time and sedentary time; body composition (adiposity), physical function and psychological well-being. *Accelerometer-based outcomes:* LiPA was calculated as 24 h – [sedentary time + standing + MVPA]. Standing time and sedentary time were derived directly from the AP3M output. *Body composition (adiposity):* weight and height were measured using an electronic scale (Seca model 770, Seca Ltd., Birmingham, UK) and stadiometer (Seca model 214, Seca Ltd., Birmingham, UK). Body mass index (BMI) was calculated, weight (kg)/height (m)^2^. Waist circumference (WC) was recorded to the nearest 0.1 cm with an adjustable anthropometric un-elastic tape (Seca model 200, Seca Ltd., Birmingham, UK). *Physical function* was assessed by the Timed Up & Go Test (TUG) and the 6-min walk test (6MWT: ATS, 2002). TUG assesses functional mobility and balance, since it is highly correlated and concurrently valid with gait speed (47). From a seated position, participants were required to stand, walk three meters, turn around, walk back and sit down, as briskly and as safely as possible. The time taken to complete the TUG test was recorded in seconds. TUG possesses high intra- and inter-rater reliability, *n* = 10–30, ICC = 0.99; ICC = 0.98 (48) and minimum detectable change is 2.08 s (47). The 6MWT is a valid measure of functional exercise capacity with stable and reproducible results (49). Participants walked as briskly and safely as possible, up and down a 30 meter straight flat track, continuously for 6 min. The distance that they covered was recorded to the nearest meter. *Psychological well-being* was assessed using the seven-item version of The Warwick-Edinburgh Mental Well-being Scale (WEMWBS) (50) which has demonstrated high correlation with other scales that measure positive mental health and well-being while it is highly sensitive to changes in mental well-being (51).

All data for primary and secondary outcomes were entered manually by two researchers, and checked for input error. Where appropriate, average scores were calculated.

### Demographic and socio-environmental measures

Questionnaires collected data on demographics (age, gender, marital status, education level, health insurance and occupational status), prevalence and type of chronic diseases (a list of 22 conditions, in accordance with the International Classification of Diseases), and on environmental conditions known to influence independent PA (perceived safety, convenience and functionality) (52).

### Data analysis

Descriptive statistics were summarised by trial arm at baseline, and reported as means and standard deviations or *n* and percentages as appropriate. Following inspection of distributional and missing data (missing at random) assumptions, we used linear mixed models to estimate the adjusted differences in means of primary and secondary outcomes between groups post-intervention and at follow up and explore differences in patterns of change over time while accounting for correlation introduced by repeated measurements.

Based on an ecological perspective of active living (52), a comprehensive set of covariates was considered (Appendix A) and each covariate examined to understand how they relate, on their own, to the initial status and rate of change of the outcomes. The LSP (Clare, Limerick), by which the randomisation was stratified, was accommodated by its inclusion as a covariate in the model. A categorical variable ‘Group’ (effect coded −1, 0, and 1 for participants in MFL, UP and CON, respectively) was tested to explore any trial arm differences in the initial status and changes over time (i.e., interaction with time). Time was modelled both as a fixed and as a random effect to account for the hierarchical structure of the data (observations “nested” within participants). Initial models also tested for nonlinear effects by including a quadratic parameter (Time × Time) in the fixed effects.

For each outcome, variables with *p*-values >0.10 in the initial models, and variables central to the research questions (e.g., Group, Time, and their interaction, Local Sports Partnership), were included in a subsequent multivariable model. We tested several covariance structures appropriate for longitudinal data (unstructured, compound symmetry, first-order autoregressive) to determine the error covariance structure that best fit the data.

The models for continuous outcome variables were calculated using the Linear Mixed Models procedure with maximum likelihood estimation in SPSS version 26, whereas the model for the categorical outcome variable “compliance with PAGL” was calculated using the Generalized Estimating Equations procedure in SPSS version 26. Analyses followed an intention-to-treat principle and all available observations were used to estimate the models. Differences in adjusted means at each time point and Group × Time interactions are presented with their corresponding 95% confidence intervals and *p*-values, which are considered mainly exploratory given the feasibility nature of this study and lack of formal sample size calculation, though numbers required to sustain each PA programme for the study period were rather large. Statistical significance was set at *p* < 0.05.

## Results

### Demographics and feasibility

#### Demographics

**Figure 1:**
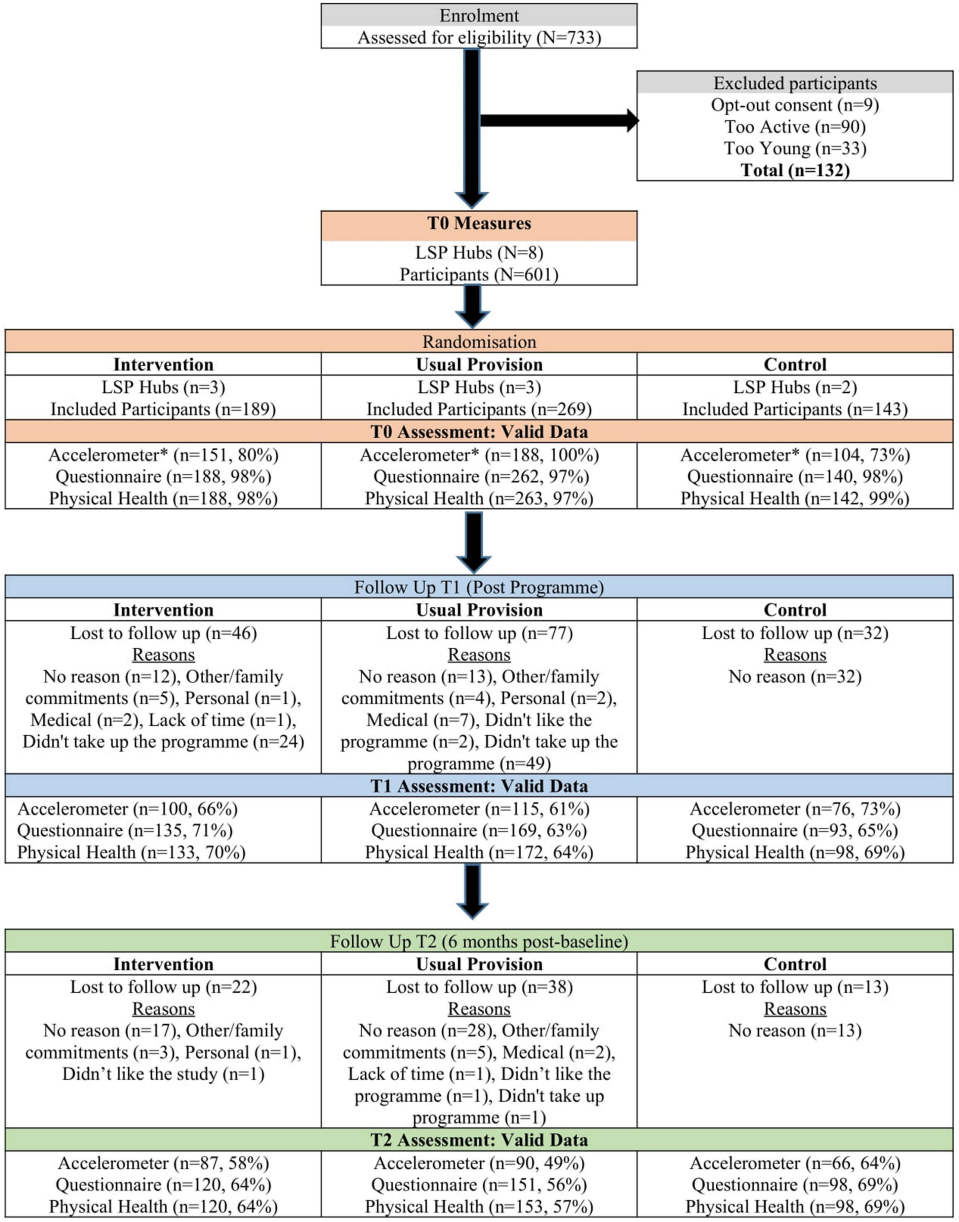
Consort flowchart of the move for life study.*Accelerometer devices were limited, thus maximum administrations adhering to study wear time protocol and within project timeline was N=443. This equated to a maximum of 188 accelerometer datasets that could be collected for each arm of the study at baseline. Only the usual provision arm reached this maximum number.

MFL recruited 733 individuals (May–September, 2018), 98% (*n* = 724) consented and completed baseline measures. As seen in Figure 1, 18% (*n* = 132) were excluded due to opt-out consent, age (aged under 50 years) or self-reported activity status (if they accumulated at least 30 min of MVPA during 5 days or more over a typical or usual week, and if 4 days or less, if they had accumulated at least 2.5 h MVPA in the last 7 days). Excluded individuals were younger (59.4 vs. 63.06, *p* < 0.001), more active (activPAL MVPA mins (10 min bouts) 32.12 vs. 13.06, *p* < 0.001) and more males (29.9% vs. 19.6%, *p* < 0.01) than included. Table 1 presents the baseline demographic characteristics of 601 included participants. Most were female (80.4%), 37% had ≥3 chronic conditions; 41% were obese. Trial arms were balanced at baseline regarding sociodemographic characteristics, with age and marital status the only significant differences between arms. UP were older than other participants, and CON were more likely to be separated or divorced. No differences were found on economic status, highest level of education or health insurance.

**Table 1:** Baseline (T0) characteristics of study participants by trial arm (*n* = 601).

	Study group		
Descriptive variables	MFL	UP	CON
Age: mean (SD), *N*	61.86 (7.98), 189	64.22 (8.51), 269	62.50 (7.37), 143
Gender: *N* (%) Male	29 (15.3)	58 (21.6)	31 (21.7)
Female	160 (84.7)	211 (78.4)	112 (78.3)
Level of education: *N* (%)			
Primary or no formal training	16 (8.7)	22 (8.4)	6 (4.3)
Lower secondary	30 (16.3)	37 (14.1)	23 (16.3)
Upper secondary	50 (27.2)	57 (21.8)	32 (22.7)
Post-secondary, non-tertiary	6 (3.3)	17 (6.5)	3 (2.1)
Non degree	35 (19.0)	65 (24.8)	44 (31.2)
Degree or higher	47 (25.5)	64 (24.4)	33 (23.4)
Medical card: *N* (%) Yes	56 (30.8)	102 (38.9)	45 (31.9)
No	126 (69.2)	160 (61.1)	96 (68.1)
Marital status: *N* (%)			
Married/living with partner	128 (69.2)	170 (64.6)	87 (61.7)
Other	57 (30.8)	93 (35.4)	54 (38.3)
Number of chronic health conditions:			
Mean (SD), *N*	2.53 (1.44), 150	2.77 (1.63), 204	2.47 (1.33), 101
Area deprivation index: *N* (%)			
Marginally below average	189 (100.0)	180 (66.9)	49 (43.3)
Disadvantaged	–	89 (33.1)	94 (65.7)
Geographical location: *N* (%)			
Rural	189 (100.0)	53 (19.7)	–
Urban	–	216 (80.3)	143 (100.0)
MVPA mins (10 min bouts) (SD), *N*	13.69 (13.95), 151	12.44 (15.44), 188	13.05 (12.87), 104

MFL = Move for Life Intervention Group, UP = Usual Provision, CON = Control. MVPA mins (10 min bouts) = MVPA minutes/day (in bouts of 10 min) (lower Ns than in other variables reflect the circumstance that accelerometers were worn by a subsample of participants).

#### Adherence

Tutor attendance logs were highly correlated with participant reported attendance rates (*r* = 0.751, *p* < 0.001). Average attendance was higher in MFL (63.8%) compared to UP (59.2%). *Fidelity:* all programme sessions were delivered per guidelines. MFL compliance was 77%, as 508 out of required 662 intervention strategies were delivered as prescribed. Twenty-seven peer mentors were identified and trained in a timely manner (exceeding progression criteria). *Retention:* The study retention rate was 63% (*n* = 374), with MFL, UP, and CON groups achieving retention rates of 64, 58 and 79%, respectively (Figure 1). Dropouts were younger (63.97 (7.8) vs. 61.59 (8.6) years; *p* < 0.001), less active (14.22 (13.4) vs. 10.83 (5.6) MVPA mins/day (in bouts of 10 min), *p* < 0.05) and more likely to be in work as opposed to retired (work 34% vs. 46%, retired 48% vs. 35%; *p* < 0.05). “No reason” was the main explanation for dropout. “Didn’t take up the programme”, followed by unrelated medical issues, no exercise training, and personal reasons. *Data Provision:* for those involved in the study at each time point valid data completion rates for accelerometers were ≥ 66%, for psychosocial questionnaires ≥89%, and for physical health assessments ≥90%. *Progression criteria:* all study progression criteria set in the study protocol (33) were met.

### Primary and secondary outcomes

The raw (unadjusted) means of the primary and secondary outcomes and number of participants at each time point in each of the comparison groups are shown in Appendix B whereas Table 2 shows the percentage change for each group (increase or decrease) in the study outcomes based on the raw means. Table 3 displays differences in adjusted means between groups at T0, T1, and T2 with corresponding *p*-values. In addition, the table shows “Group × Time” interaction coefficients and corresponding *p*-values, examining variation in study outcomes over time (T0, T1, T2) as a function of group membership/treatment condition (MFL, UP, CON). To interpret “Group × Time” interactions and assess intervention effects, in addition to the coefficient of interaction terms, we used visualizations (graphs) spanning the entire range of possible values for the Y axis (53). Missing observations for participants included in the analyses ranged from 23% (accelerometer-based variables) to 27% (other variables).

**Table 2:** Percentage change in outcome variables according to study group and period.

		T0-T1			T0-T2	
	MFL	UP	CON	MFL	UP	CON
MVPA	−8.7%	0.1%	−23.3%	−21.7%	2.9%	−24.5%
LiPA	−3.3%	3.8%	−2.1%	−5.6%	1.5%	−10.6%
Stand	−1.8%	5.6%	5.3%	−2.0%	−1.6%	−3.5%
Sed. Time	0.8%	−1.9%	−0.2%	0.7%	−1.5%	4.8%
PAGL	95.4%	68.5%	23.4%	85.4%	69.0%	22.4%
BMI	−3.1%	−0.3%	−0.7%	−2.3%	−0.9%	−0.1%
Waist circ.	−6.6%	−2.0%	−3.3%	−6.2%	−3.6%	−3.4%
TUG	−10.9%	0.9%	−7.9%	−9.3%	−0.4%	−6.6%
6MWT	8.5%	6.9%	2.5%	10.6%	8.1%	3.4%
Well-being	2.6%	0.92%	1.3%	3.2%	1.2%	−1.1%

MFL = Move for Life Intervention Group, UP = Usual Provision, CON = Control; MVPA = moderate to vigorous physical activity, Stand = standing time, LiPA = light physical activity, Sed. Time = sedentary time during waking hours, PAGL = compliance with physical activity guidelines, BMI = Body Mass Index. Waist Circ. = waist circumference, TUG = Timed Up & Go Test, 6MWT = Six-Minute Walk Test; percentage changes are based on raw (unadjusted) values.

**Table 3:** Group differences over time in outcome variables.

	Adjusted mean difference (95% CI) T0	*p*-value	Adjusted mean difference (95% CI) T1	*p*-value	Adjusted mean difference (95% CI) T2	*p*-value	Group × Time interaction (95% CI)	*p*-value
**MVPA (min)**								
MFL vs. Control	2.94 (−2.23, 8.10)	0.264	4.14 (−0.82, 9.10)	0.101	6.57 (0.79, 12.35)	0.026	8.40 (−1.67, 18.47)	0.102
MFL vs. UP	5.58 (1.04, 10.13)	0.016	4.05 (−0.34, 8.45)	0.070	3.50 (−1.59, 8.58)	0.177	−1.00 (−9.72, 7.72)	0.821
UP vs. Control	−2.65 (−7.60, 2.31)	0.294	0.09 (−4.68, 4.85)	0.971	3.07 (−2.47, 8.61)	0.276	9.40 (−0.23, 19.04)	0.056
**LiPA (hours)**								
MFL vs. Control	0.11 (−0.02, 0.24)	0.090	0.13 (0.01,0.25)	0.040	0.14 (0.02, 0.26)	0.018	0.04 (−0.01, 0.08)	0.116
MFL vs. UP	0.14 (0.03, 0.25)	0.012	0.14 (0.03–0.25)	0.010	0.15 (0.05, 0.25)	0.004	0.01 (−0.04, 0.05)	0.778
UP vs. Control	−0.03 (−0.15, 0.09)	0.605	−0.01 (−0.13,0.10)	0.834	−0.01 (−0.12, 0.10)	0.898	0.03 (−0.01, 0.08)	0.181
**Stand (hours)**								
MFL vs. Control	0.82 (0.23, 1.42)	0.007	0.81 (0.22, 1.40)	0.008	0.50 (−0.12, 1.12)	0.111	−0.69 (−1.35, −0.04)	0.037†
MFL vs. UP	0.95 (0.46, 1.43)	<0.0001	0.96 (0.48, 1.44)	<0.0001	0.77 (0.25, 1.28)	0.004	−0.39 (−0.96, 0.18)	0.183
UP vs. Control	−0.12 (−0.49, 0.24)	0.500	−0.15 (−0.49, 0.19)	0.380	−0.26 (−0.66, 0.13)	0.191	−0.31 (−0.93, 0.32)	0.334
**Sed. time (hours)**								
MFL vs. Control	−0.23 (−0.66, 0.19)	0.284	−0.32 (−0.74, 0.10)	0.132	−0.41 (−0.80, 0.02)	0.039	−0.22 (−0.45, 0.01)	0.062
MFL vs. UP	−0.64 (−1.01, −0.27)	0.001	−0.61 (−0.96, −0.26)	0.001	−0.60 (−0.93, −0.26)	0.001	0.06 (−0.15, 0.27)	0.595
UP vs. Control	0.41 (−0.003, 0.82)	0.052	0.29 (−0.11, 0.69)	0.152	0.18 (−0.19,0.56)	0.339	−0.28 (−0.50, −0.05)	0.017†
**PAGL** ^**a**^ **(%)**								
MFL vs. Control	1.0 (−13.0, 15.0)	0.880	15.0 (5.0, 26.0)	0.003	25.0 (14.0, 36.0)	<0.0001	12.97 (3.27, 51.51)	<0.0001†
MFL vs. UP	7.0 (−4.0, 18.0)	0.193	12.0 (4.0, 20.0)	0.003	14.0 (6.0, 22.0)	<0.0001	3.83 (1.09, 13.48)	0.036†
UP vs. Control	−6.0 (−17.0, 5.0)	0.298	3.0 (−6.0, 13.0)	0.514	11.0 (0.00, 23.0)	0.054	3.38 (1.07, 10.70)	0.038†
**BMI**								
MFL vs. Control	1.96 (0.54, 3.39)	0.007	1.97 (0.54, 3.40)	0.007	2.00 (0.58, 3.43)	0.006	0.05 (−0.10, 0.19)	0.524
MFL vs. UP	1.22 (−0.02, 2.46)	0.054	1.27 (0.02, 2.51)	0.047	1.32 (0.08, 2.56)	0.037	0.12 (−0.01, 0.25)	0.076
UP vs. Control	0.74 (−0.61, 2.09)	0.282	0.70 (−0.66, 2.06)	0.310	0.68 (−0.67, 2.03)	0.322	−0.07 (−0.21, 0.68)	0.318
**Waist circ. (cm)**								
MFL vs. Control	8.90 (4.86, 12.95)	< 0.0001	8.55 (4.53, 12.57)	<0.0001	7.11 (3.02, 11.19)	0.001	−3.11 (−5.65, −0.57)	0.017†
MFL vs. UP	6.21 (3.03, 9.38)	0.0001	5.27 (2.13, 8.41)	0.001	3.67 (0.47, 6.88)	0.025	−4.63 (−6.81, −2.46)	<0.0001†
UP vs. Control	2.69 (−0.78, 6.17)	0.128	3.28 (−0.16, 6.72)	0.061	3.44 (−0.08, 6.95)	0.055	1.52 (−0.89, 3.94),	0.216
**TUG (sec)**								
MFL vs. Control	−0.32 (−0.85, 0.20)	0.227	−0.41 (−0.93, 012)	0.126	−0.52 (−1.05, 0.02)	0.058	−0.38 (−0.95, 0.20),	0.199
MFL vs. UP	−0.27 (−0.71, 0.18)	0.245	−0.53 (−0.97, −0.08)	0.020	−0.85 (−1.30, −0.39)	<0.0001	−1.08 (−1.58, −0.58)	<0.0001†
UP vs. Control	−0.06 (−0.37, 0.25)	0.711	0.12 (−0.16, 0.40)	0.400	0.33 (0.01, 0.66)	0.045	0.71 (0.16 1.26)	0.012†
**6MWT (meters)**								
MFL vs. Control	12.57 (−14.57, 39.70)	0.363	16.90 (−10.03, 43.84)	0.218	23.81 (−2.25, 49.88)	0.073	14.09 (5.20, 22.99),	0.002†
MFL vs. UP	31.81 (9.06, 54.57)	0.006	30.67 (8.03, 53.32)	0.008	32.80 (10.97, 54.63)	0.003	1.24 (−6.61, 9.08)	0.756
UP vs. Control	−19.24 (−35.25, −3.24),	0.019	−13.77 (−28.43, 0.89)	0.066	−8.99 (−23.33, 5.35)	0.299	12.85 (4.42, 21.29)	0.003†
**Well-being**								
MFL vs. Control	−0.89 (−1.82, 0.03)	0.059	−0.62 (−1.46, 0.22)	0.148	−0.38 (−1.16, 0.41)	0.345	0.61 (0.12, 1.09)	0.014†
MFL vs. UP	0.048 (−0.75, 0.84)	0.905	0.25 (−0.49, 0.98)	0.515	0.36 (−0.34, 1.06)	0.316	0.36 (−0.06, 0.79)	0.092
UP vs. Control	−0.94 (−1.81, −0.07)	0.035	−0.86 (−1.66, −0.07)	0.032	−0.74 (−1.47, 0.004)	0.051	0.24 (−0.24, 0.72)	0.323

MFL = Move for Life Intervention Group, UP = Usual Provision Group, CON = Control Group; MVPA = moderate to vigorous physical activity, LiPA = light physical activity, Stand = standing time, Sed. Time = sedentary time (during waking hours), PAGL = physical activity guidelines, BMI = body mass index, Waist Circ. = waist circumference, TUG = Timed Up & Go Test, 6MWT = Six-Minute Walk Test. ^†^Significant “Group × Time” interaction indicative of intervention effects. Models are adjusted by Local Sports Partnership and relevant demographic, biological, social, and environmental variables.

a Estimated difference in percentage of participants meeting physical activity guidelines.

#### Accelerometer-based variables and self-reported compliance with activity guidelines

**Figure 2:**
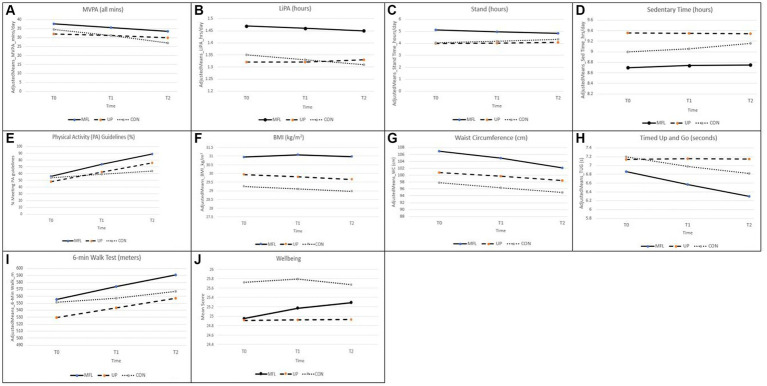
**(A–J)** Changes in accelerometer determined MVPA **(A)**, LiPA **(B)**, stand **(C)**, and sedentary time **(D)**, in proportion meeting PA guidelines **(E)**, in anthropometric measures [BMI **(F)** and waist circumference **(G)**] and in physical function measures [timed up and go **(H)** and 6-min walk test **(I)**] and well-being **(J)**.

As shown in Table 3, we found a significant “Group × Time” interaction for self-reported compliance with PAGL (whole sample) and for accelerometer-determined standing time and sedentary time (subsample wearing accelerometers). As illustrated in the corresponding visual graph (Figure 2E), compliance with PAGL increased over time in the three study groups. Furthermore, compliance with PAGL increased significantly in the MFL group relative to both UP and CON and in UP relative to CON. Notably, the adjusted mean differences, shown in Table 3, reveal that the percentage of participants who reported meeting PAGL was 15 and 12 points higher in MFL at T1, and of 25 and 14 points at T2, compared to participants in CON and UP, respectively (all *p* < 0.01). In addition, compared to CON, 11% more UP participants reported meeting PAGL at T2, with the difference approaching the specified level of statistical significance (*p* = 0.054).

Although the difference in adjusted means in accelerometer-derived MVPA between MFL and CON participants was quite large at T2 (6.57 min/day, *p* = 0.026), the “Group × Time” interaction coefficient was not significant in any of the comparisons involving the three study groups (Table 3). This suggests that there were no intervention effects regarding this outcome for the sub-sample wearing accelerometers. A similar conclusion is applicable to LiPA. On the other hand, we observed a significant “Group × Time” interaction for accelerometer-based standing time and sedentary time. Specifically, the interaction coefficients and corresponding graphs (Figures 2C,D) indicate that standing time decreased in MFL relative to the CON group while sedentary time increased in the latter compared to UP. In addition to the figures, the differences in adjusted means at T1 and T2 are suggestive of relatively modest interaction effect sizes, particularly for standing time.

#### Body composition (adiposity)

Whilst we did not find evidence of intervention effects in the form of significant “Group × Time” interactions for BMI, we observed such evidence for waist circumference. In particular, the interaction coefficients and corresponding graph (Figure 2G) indicate that even though waist circumference remained higher in the MFL group and decreased, as seen in Table 2, in the three groups from T0-T2 (−6.2% MFL, −3.6% UP, −3.4% CON), it also decreased in MFL relative to both UP (*p* < 0.0001) and CON (*p* < 0.017), as shown in Table 3.

#### Physical function

TUG scores decreased in the MFL group relative to UP, indicating that test scores improved significantly over time in MFL participants compared to UP participants. Specifically, the former outperformed significantly the latter by −0.53 s (*p* = 0.020) at T1 and − 0.85 s at T2 (*p* < 0.0001). In addition, CON participants outperformed UP participants over time, as evidenced in the significant “Group × Time” interaction coefficient for these two groups (Table 3; Figure 2H). This was particularly true at T2, where CON participants took on average 0.33 s less to complete the test than UP participants (*p* = 0.045).

Finally, even though adjusted mean differences did not reach statistical significance neither at T1 nor at T2, both MFL and UP increased significantly the distance covered in the 6MWT relative to CON over the study period, as evidenced in the “Group × Time” interaction coefficients in Table 3 and the corresponding graph (Figure 2I). Illustrating this circumstance, from T0-T2, the MFL, UP and CON participants increased on average by 10.6, 8.1 and 3.4%, respectively (Table 2).

#### Psychological well-being

A significant “Group × Time” interaction coefficient (Table 3) together with the temporal pattern of adjusted mean differences and the corresponding graph (Figure 2J) indicate that psychological well-being increased significantly in MFL relative to CON during the study period. Specifically, as seen in Table 2, from T0-T2, well-being scores increased on average by 3.2% in MFL participants and decreased by 1.1% in CON participants. Meanwhile, UP participants reported a small increase of 1.2%.

## Discussion

This study aimed to establish the feasibility of the MFL intervention and its potential for improving activity related energy expenditure and associated health outcomes. The results provide evidence of the promise and added value of the MFL augmentation to enhance current community PA programmes for adults aged 50 years and over in Ireland. Developing effective interventions for inactive and older adults is especially important to maximise public health goals for NCD prevention (54). The learnings from the MFL trial and its delivery of intervention were maximised by combining feasibility and promising evidence (55, 56), while our results showed that progression criteria were met in an acceptable timeframe (57).

MFL is a theory-informed, pragmatic, community-based intervention to promote PA for inactive adults aged 50 years or older. MFL drew on both traditional evidence-to-practice and complementary practice-to-evidence pathways for its design (30, 58). Its conceptualisation allowed for the provision of instructional technical skills to remain within the professional model, but informed by formative research (40), MFL added training on behavior change strategy use and methods for enhancement of group dynamics to the tutor skillset. It also trained peer mentors to improve in delivery, organisational capacity and provide participant encouragement for long-term PA adherence both during and after the intervention. The findings from this study add to the evidence showing that approaches based on enlisting peer volunteering support have potential to be an effective strategy for increasing PA in adults and older adults, particularly those who are inactive and socially disengaged (27).

The study methods were acceptable for both LSPs and inactive middle aged and older adults, evidenced in its co-creation with the LSPs and the participants themselves (40), as research recommends (59); and also in its successful recruitment of LSP tutors and peer mentors across 32 PA programmes showing high level of interest in this type of professional and personal education. Zubala et al. published a review (60) that called for future research on the promotion of PA to community-dwelling older adults to emphasize systemic and contextual factors. By anchoring the MFL programme within the LSP network we ensured it would benefit from the vast local structures increasing its likelihood of becoming embedded within a community organisation, minimising barriers to implementation at the organisational level (30). The circumstance that the MFL intervention programme was proposed as an ‘augmentation’ aiming to enrich existing LSP programmes, instead of as being a new programme, was a key asset in this regard (36).

The study met all progression criteria. Recruitment of individuals to intervention trials can be expensive, time consuming and problematic, particularly when targeting inactive older adults (61). Our ambitious recruitment targets (33) were met and indeed exceeded, and 82% of individuals who declared an interest met the study inclusion criteria. Findings showed that service providers can maximise the reach and recruitment for community-based health promotion initiatives through partnership-based recruitment strategies anchored within community groups. However, significantly fewer males than females took part, reflecting the difficulty in recruiting males to health promotion community-based research studies (62–64). Future recruitment and assessment strategies as well as PA programmes offered need to be gender sensitised.

In Ireland, 61% of adults aged 50 years or more live with a chronic condition such as arthritis, osteoporosis, cardiovascular diseases (65). Tackling obesity and weight gain presents a major national issue (66), with 43% of those aged 50 years or more to be overweight and 36% obese (67). At baseline, all participants were inactive, 82% reported living with at least one chronic condition, 40% were overweight and 41% were obese, showcasing the need for interventions of this nature for this target group.

This study demonstrated that research required to evaluate the effectiveness of the intervention in a larger trial is feasible. In accordance with the study progression criteria (33), and considering the population (inactive, older adults) the retention rate exceeded its 60% target, at 63%. The control group had the highest retention rate (79%), reflected in other studies (68), demonstrating a potential untapped interest in ‘assessment only’ interventions. Questionnaire, physical health and accelerometer data provision were very high at baseline (>98%), however it decreased overtime. Physical health data completion rates exceeded questionnaire, potentially indicating a preference for physical health measures by participants. Although progression criteria (>40%) were met, study retention was low. A main reason for dropout was the fact that participants ‘did not take up the programme’, highlighting a feasibility issue, as some participants did not like the type, time of day or day/week on which the PA programmes were offered and consequently chose not to receive the programme. MFL was a complex intervention involving two LSPs, eight hubs and 32 PA programmes and yet it did not meet the needs of programme choice for all participants, particularly those who were younger, still in work and very inactive. A definitive trial could consider a ‘home-based’ or online option to broaden accessibility.

The results provide promising evidence that the MFL intervention to improve PA and associated health outcomes. While we found relatively small, but significant, differences among groups regarding device-based standing time and sedentary time favouring UP in the subsample wearing accelerometers, in the whole sample, self-reported compliance with PAGL was largely favourable to MFL compared to both UP and CON. As mentioned earlier, post-intervention, the percentage of participants who reported meeting PAGL was 15 and 12 points higher in MFL compared to participants in CON and UP, respectively, while this difference increased to 25 and 14 points at follow up. This finding is noteworthy when compared to other PA interventions in similar settings and populations (21, 60), particularly considering that MFL is a relatively low dose ‘augmentation’ of existing PA programmes. Furthermore, the findings regarding self-reported compliance with PAGL are largely consistent with the findings regarding body composition (waist circumference), physical function (TUG and 6MWT) and psychological well-being in the whole sample, which favoured the MFL group overall. As a case in point, waist circumference decreased in MFL relative to both UP and CON over the study period. Considering the relatively short study period, relatively low intervention dose, and the health consequences associated with markers of adiposity (69), the average decrease of 6.11 cm from baseline to 6-month follow up in the MFL group is remarkable compared to reductions in waist circumference documented in other adult PA interventions for adults (70). Moreover, the observed decrease is seemingly commensurate with the pattern of self-reported compliance with PAGL. One circumstance that may explain partially the magnitude of changes observed is that MFL participants had higher average initial waist circumference values than UP and CON participants and, therefore, more room for improvement and, perhaps, greater motivation to do so. Similarly, MFL was also the group displaying higher baseline levels of MVPA, LiPA and standing time and lower sedentary time, which may have negatively influenced to a certain degree its trajectory regarding accelerometer-determined PA and sedentary time. Taken together, improvements (absolute and relative to other groups) in compliance with PAGL, body composition (waist circumference), functional mobility and balance (TUG) and functional exercise capacity (6MWT) help to explain the significant, although relatively modest, increase in psychological well-being scores observed in MFL relative to CON over a 6-month period.

While MFL participants may have overestimated their PA when providing self-assessments, measurement reactivity amongst the subsample that wore accelerometers may also have been present (71). It is also possible that some of the observed differences were due to accelerometers not capturing activities that participants may have performed more often as a result of their involvement in the organised programmes (e.g., cycling).

Achieving long-term adherence to exercise for older adults requires theoretically informed interventions (72), yet there is insufficient evidence of long-term improvements (21), and indication that after 20 weeks following a programme PA behavior returns near to baseline levels (73). The findings regarding self-reported compliance with PAGL show the potential for sustainability of intervention effects in the MFL group vis-à-vis levels of PA at 6-month follow up. Given the growing evidence indicative of the health benefits of LiPA and reduced sedentary time (74), the significant results concerning accelerometer-based standing time and sedentary time are also encouraging for established community-based PA programmes for adults delivered via LSPs in Ireland. In this vein, UP also showed significant improvements over time in self-reported compliance with PAGL relative to the CON group, although smaller in magnitude than MFL at each time point considered, and similar advantages over time than MFL in the 6MWT compared to CON. For progress to occur in PA promotion, more rigorous evaluation studies of real-world programmes is needed (30, 58), particularly since practice moves faster than research (75). The present study aimed to address this call by providing a rigorous evaluation of existing public community PA programmes in Ireland in addition to providing evidence of effectiveness for the MFL augmentation.

The real-world context and pragmatic approach of this research conferred both strengths and limitations. The development of a partnership network involving the statutory, academic and community sectors was key to the MFL model development and subsequent delivery. The results showed that a ‘real world’ community-based PA programme can work under usual conditions (76), and enable those previously inactive to achieve, and sustain, significant improvements. Other notable strengths include high number of participants recruited and the comprehensive set of evaluation measures to assess primary and secondary outcomes. Finally, our analytical strategy took into account important aspects of the complexity of the study design and variation in PA behavior, such as the hierarchical nature of data, change over time, linear and non-linear effects, and covariates representing multiple domains and levels of influence.

Any further research aimed at building upon this trial will need to address some specific limitations. While this study has strong external validity and generalisability and consequently applicability of findings to real-life contexts, thus strengthening the likelihood of intervention sustainability and scalability, however, some aspects of internal validity may have been compromised. The recruitment of males was challenging; gender sensitised recruitment, testing and programme options are required. As the potential recruitment was unknown, a strategy that encouraged people to register in advance, without excluding anyone, led to variation in numbers attending testing sessions. Consequently, over recruitment occurred is some hubs, and understaffing on some initial testing nights. While this affected only baseline, future recruitment strategies should aim for a more robust registration procedure. The limitations of count-to-activity thresholds for the determination of activity intensities should also be acknowledged. Within the literature, there is a scarcity of validation studies conducted in free-living environments (77) and the current prediction analysis techniques that use count-to-activity thresholds can result in under or over-estimations (78). However, no consensus on the “correct” count-to-activity threshold exists. Those used to determine MVPA from the activPAL in this study had high sensitivity (95%) and specificity (89%) values, indicating that they are accurate at detecting both the activities above a specific threshold (i.e., 3 METs), and also are below the said threshold, while they have been previously been applied to an older adult population (79). The total study period consisted of the time from baseline to 6-month follow-up, this falls short of the minimum 6-month follow up period post programme, recommended in widely used planning and evaluation frameworks, such as RE-AIM (80).

## Conclusion

Overall, the findings show that the MFL intervention is feasible. Considering the characteristics of the target population (i.e., middle age to older, inactive adults, high prevalence of obesity and comorbidities) and the pattern of results for outcomes related to energy expenditure, body composition, physical function, and well-being, the intervention has potential to enhance current community PA programmes for adults aged 50 years and over. A full effectiveness and cost-effectiveness pragmatic trial is warranted.

## Data availability statement

The raw data supporting the conclusions of this article will be made available by the authors, without undue reservation.

## Ethics statement

The studies involving humans were approved by University of Limerick, Faculty of Education and Health Sciences Research Ethics Committee (registration no. 2018_02_15_EHS; 09 April 2018). The studies were conducted in accordance with the local legislation and institutional requirements. The participants provided their written informed consent to participate in this study.

## Author contributions

CW: Conceptualization, Data curation, Formal analysis, Funding acquisition, Investigation, Methodology, Project administration, Supervision, Validation, Writing – original draft, Writing – review & editing. AO'R: Conceptualization, Methodology, Writing – review & editing. CD: Methodology, Project administration, Supervision, Writing – original draft, Writing – review & editing. GH: Data curation, Formal analysis, Methodology, Project administration, Software, Validation, Writing – review & editing. AC: Conceptualization, Funding acquisition, Methodology, Writing – review & editing. AD: Conceptualization, Funding acquisition, Methodology, Writing – review & editing. PG: Formal analysis, Methodology, Writing – review & editing. LG: Conceptualization, Funding acquisition, Methodology, Supervision, Writing – review & editing. AM: Conceptualization, Funding acquisition, Methodology, Supervision, Writing – review & editing. AS: Data curation, Formal analysis, Investigation, Methodology, Software, Writing – review & editing. EB: Conceptualization, Data curation, Formal analysis, Investigation, Methodology, Project administration, Supervision, Validation, Writing – original draft, Writing – review & editing.

## Footnotes

^1^ https://www.who.int/news-room/fact-sheets/detail/ageing-and-health
