# Supplementary material for: Move for Life an intervention for inactive adults aged 50 years and older: a cluster randomised feasibility trial
Source: Front Public Health. 2024 May 15;12:1348110. doi: 10.3389/fpubh.2024.1348110 (PMC11133700; doi:10.3389/fpubh.2024.1348110)
Supplement: Supplementary file 1 [file Table_1.DOCX]

**Additional File 1: Table 1.** Details of covariate measurement and responses.

| **Covariate** | **Purpose of Measure** | **Question asked** | **Responses included in analysis*** |
| --- | --- | --- | --- |
| Age | Determine age of participant. | Age | Years |
| Gender | Determine the gender of each participant. | Gender | 0 = Male or 1 = Female |
| Education | Determine the highest level of education achieved by each participant. | What is your highest level of education? | 1 = primary or no formal training; 2 = lower secondary; 3 = upper secondary; 4 = post-secondary non tertiary; 5 = non degree; 6 = degree or higher.  Used as a continuous measure in the analysis. |
| Medical card | A medical card provides access to medical services, prescription medicines and hospital care for free. This was used as a proxy measure for socio-economic status. | Do you have a medical card? | -1 = Yes or 1 = No |
| Marital status | Determine the marital status of each participant. | What is your current marital status? | 1 = married/living with partner; 2 = widowed; 3 = separated/divorced; 4 = single/never married  Recoded as: 0 = married/living with partner or 1 = other |
| Number of health conditions | Determine the number of health conditions each participant has currently or in the past. | Ever told had 1) Arthritis, 2) Previous fractures, 3) Osteoporosis, 4) High blood pressure/hypertension, 5) Angina, 6) Congestive heart failure ,7) Heart attack, 8) Stroke, 9) Mini stroke/TIA, 10) High cholesterol, 11) Heart murmur, 12) Abnormal heart rhythm, 13) Heart trouble, 14) Diabetes or high blood sugar, 15) Chronic lung disease, 16) Asthma, 17) Cancer, 18) Mental illness, 19) Parkinsons, 20) Alzheimers, 21) Dementia, 22) Memory impairment | 1 = No or 2 = Yes  Number of health conditions combined to create an overall number.  Used as a continuous measure in the analysis. |
| Area Deprivation Index | Determine the area socioeconomic disadvantage. | POBAL deprivation index score: Government of Ireland. (www.pobal.ie). | -1 = Marginally below average or 1 = Disadvantage |
| Geographical Location | Urban  Rural | Population density > 25,000  Population density <6,500 | 0 = Urban or 1= Rural |
| Access to public transport | Determine how easy or difficult it is for participants to access public transport from their house. | How easy, or difficult, is it to use public transport near your house? | 1 = very easy; 2 = somewhat easy; 3 = neither easy nor difficult; 4 = difficult; 5 = very difficult.  Used as a continuous measure in the analysis. |
| Perceived safety day | Rate each participants perception of safety during the day. | How safe do you feel “out and about” during the day? | 1 = very safe; 2 = fairly safe; 3 = neither safe nor unsafe; 4 = fairly unsafe; 5 = very unsafe  Used as a continuous measure in the analysis. |
| Perceived safety night | Rate each participants perception of safety during the night. | How safe do you feel “out and about” during the night? | 1 = very safe; 2 = fairly safe; 3 = neither safe nor unsafe; 4 = fairly unsafe; 5 = very unsafe  Used as a continuous measure in the analysis. |
| Perceived walkability | Rate each participants perception of their neighbourhoods walkability. | Overall, how would you rate your neighbourhood as a place to walk? | 1 = very walkable; 2 = somewhat walkable; 3 = neither walkable nor unwalkable; 4 = not very walkable; 5 = not walkable at all  Used as a continuous measure in the analysis. |
| Local Sports Partnership | Limerick LSP  Clare LSP | Random assignment to LSP | 0=Limerick; 1 =Clare |
| Body Mass Index (BMI) | BMI measurements help classify the weight of an individual as underweight, normal,  overweight or obese. | Height measurement using standard collapsible portable stadiometer.  Weight measurement using standard portable calibrated scales (mechanical). | BMI calculated using the equation:  BMI = Weight (kg) / Height (m2)  Used as a continuous measure in the analysis. |

* No response and missing data were removed and not used in any analysis.
