# Supplementary material for: Move for Life an intervention for inactive adults aged 50 years and older: a cluster randomised feasibility trial
Source: Front Public Health. 2024 May 15;12:1348110. doi: 10.3389/fpubh.2024.1348110 (PMC11133700; doi:10.3389/fpubh.2024.1348110)
Supplement: Supplementary file 2 [file Table_2.DOCX]

*Table 2. Unadjusted values of outcome variables across the study groups at baseline (T0), time 1 (T1) and time 2 (T2) (mean (SD); n)*

|  | **Time Point 0 – Study Group** | | | **Time Point 1 – Study Group** | | | **Time Point 2 – Study Group** | | |
| --- | --- | --- | --- | --- | --- | --- | --- | --- | --- |
|  | **MFL** | **UP** | **CON** | **MFL** | **UP** | **CON** | **MFL** | **UP** | **CON** |
| MVPA  (min.) | 36.01 (20.2);  150 | 28.54 (19.7);  186 | 33.54 (19.1);  104 | 32.88 (19.8);  99 | 28.57 (21.5); 115 | 25.74 (16.6); 76 | 28.20 (18.5);  89 | 29.37 (19.8);  90 | 25.32 (15.5);  66 |
| LiPA  (hours) | 1.53 (0.5);  150 | 1.31 (0.5);  188 | 1.41 (0.5);  104 | 1.48; (0.5);  99 | 1.36 (0.4);  115 | 1.38 (0.4);  76 | 1.44 (0.4);  90 | 1.33 (0.4);  88 | 1.26 (0.4);  66 |
| Stand  (hours) | 4.96 (1.4);  151 | 4.27 (1.3);  188 | 4.56 (1.3);  104 | 4.87 (1.5);  100 | 4.51 (1.3);  115 | 4.80 (1.2);  76 | 4.86 (1.2);  90 | 4.20 (1.3);  89 | 4.40 (1.3);  66 |
| Sed. Time (hours) | 8.65 (1.6);  151 | 9.44 (1.7);  188 | 9.00 (1.7);  104 | 8.72 (1.6);  99 | 9.26 (1.6);  115 | 8.98 (1.4);  76 | 8.71 (1.5);  90 | 9.30 (1.5);  89 | 9.43 (1.5);  66 |
| PAGL (%; n) |  |  |  |  |  |  |  |  |  |
| *Active* | 43.9; 83 | 43.8; 114 | 51.4; 72 | 85.8; 115 | 73.8; 124 | 63.4; 59 | 81.4; 96 | 74.0; 108 | 62.9; 61 |
| *Insufficient* | 56.1; 106 | 56.2; 146 | 48.6; 68 | 14.2; 19 | 26.2; 44 | 36.6; 34 | 18.6; 22 | 26.0; 38 | 37.1; 36 |
| BMI | 30.19 (6.1);  188 | 29.37 (5.4);  261 | 28.91 (5.5);  142 | 29.25 (5.6);  133 | 29.28 (5.5);  172 | 28.72 (5.2);  97 | 29.51 (5.6);  120 | 29.12 (5.1);  152 | 28.88 (5.5);  98 |
| Waist Circ.  (cm.) | 98.88 (15.8); 189 | 96.23 (13.9);  263 | 94.78 (14.2);  141 | 92.31 (15.5);  133 | 94.30 (13.2);  169 | 91.67 (14.1);  97 | 92.77 (15.4);  120 | 93.33 (12.7);  151 | 91.59 (15.0);  98 |
| TUG  (sec.) | 7.24 (1.6);  186 | 6.79 (1.6);  262 | 6.49 (1.2);  142 | 6.45 (1.3);  132 | 6.85 (1.8);  170 | 5.98 (1.2);  97 | 6.57 (1.5);  120 | 6.76 (1.6);  148 | 6.06 (1.1);  98 |
| 6MWT  (meters) | 539.32 (77.6);  184 | 534.96 (91.1);  255 | 571.51 (64.4);  141 | 585.11 (71.3);  122 | 572.00 (80.4);  146 | 585.77 (60.7);  94 | 596.26 (73.9);  103 | 578.51 (87.4);  131 | 590.72 (64.3);  87 |
| Well-being  (Score: 7-35) | 24.65 (4.47);  181 | 25.08 (4.48);  254 | 25.66 (4.63);  134 | 25.30 (4.26);  135 | 25.31 (4.30);  166 | 26.00 (4.04);  93 | 25.44 (4.04);  120 | 25.37 (4.1);  151 | 25.37 (4.09);  98 |

MFL = Move for Life Intervention Group, UP = Usual Provision, CON = Control; MVPA = moderate to vigorous physical activity, LiPA = light physical activity, Sed. Time = sedentary time during waking hours, PAGL = physical activity guidelines, BMI = Body Mass Index. Waist Circ. = waist circumference, TUG = Timed Up & Go Test, 6MWT = Six-Minute Walk Test.
